# Transforming hosts into nurseries and nutrients: strategic manipulation by endoparasitoid wasps

**DOI:** 10.1016/j.jare.2025.11.012

**Published:** 2025-11-09

**Authors:** Wenxian Wu, Yueyue Liu, Song Chen, Hongling Liu, Yatao Zhou, Yanni Tan, Xing Zheng, Xu Liu, Limei He, Yi Cai

**Affiliations:** a Institute of Urban Agriculture, Chinese Academy of Agricultural Sciences, Chengdu Agricultural Science and Technology Center, Chengdu 610000 Sichuan Province, China; b Sichuan Academy of Agricultural Sciences, Chengdu 610000 Sichuan Province, China; c College of Life Science, Sichuan Agricultural University, Ya’an 625000 Sichuan Province, China

**Keywords:** Endoparasitoids, Virulence factors, Host immune suppression, Developmental manipulation, Nutrient exploitation

## Abstract

Parasitoid wasps, shaped by ecological pressures and interspecific competition, have evolved sophisticated molecular strategies to manipulate host physiology, ensuring successful parasitism and optimal progeny development. Despite substantial advances in parasitoid–host interaction research, a systematic synthesis of these findings is still lacking. This review aims to consolidate recent findings on the molecular mechanisms by which endoparasitoids regulate host physiology, and to explore their potential applications in sustainable pest management. We reviewed recent progress on virulence factors derived from venom, polydnaviruses, and teratocytes, focusing on their roles in immune suppression, developmental regulation, and metabolic reprogramming of insect hosts. Three principal mechanisms underlying parasitoid-mediated host manipulation are identified: (1) Immune suppression: Parasitoids reduce hemocyte abundance, disrupt hemocyte mobilization to impede encapsulation, inhibit phenoloxidase activation to prevent melanization, and interfere with immune signaling pathways. Some species also adopt passive immune evasion strategies. (2) Developmental regulation: Parasitoids delay host growth and metamorphosis by downregulating ecdysteroid titers, inhibiting cell proliferation, degrading host tissues, and repressing protein synthesis. (3) Metabolic reprogramming: Parasitoids manipulate host amino acid, carbohydrate, and lipid metabolism to mobilize and redirect nutritional resources toward their developing offspring. This review offers a comprehensive overview of the molecular tactics employed by endoparasitoids to modulate host physiology. By elucidating these mechanisms, we propose parasitoid-derived virulence factors as promising leads for developing novel, sustainable pest control strategies.

## Highlights

• Summarizes advances in how endoparasitoid wasps use virulence factors to manipulate host physiology.
• Provides a framework for understanding host manipulation and parasitoid–host interactions.
• Highlights parasitoid virulence factors as potential eco-friendly bioinsecticidal agents.

## Graphical abstract

**Figure:**
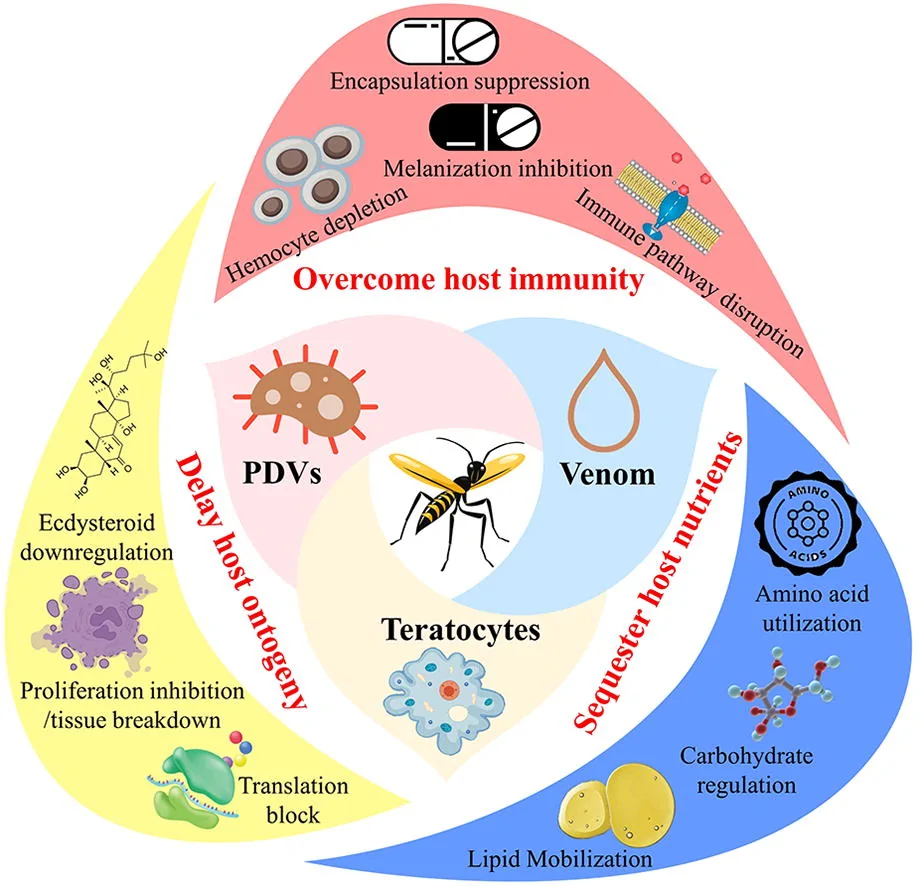

## Introduction

Endoparasitoid wasps, belonging to the order Hymenoptera, are natural enemies of arthropods characterized by free-living adults and larval stages that develop internally within their hosts, ultimately resulting in host mortality. Hymenopteran parasitoids constitute one of the most taxonomically diverse groups globally, with over 100,000 described species [48]. However, the actual diversity is likely underestimated due to the small size and inconspicuous populations of many taxa [14]. This diverse group exhibits extraordinarily host range breadth, parasitizing myriad insect orders and other arthropods [14]. Moreover, parasitoids exploit hosts across all developmental stages, from eggs to adults, demonstrating exceptional life history plasticity [80]. Parasitoid-induced mortality arises through multiple pathways, including reproductive parasitism, host feeding, and other non-reproductive effects, with considerable impacts on the host population [1]. Thus, parasitoid wasps are considered crucial natural regulators of insect pest populations, with indispensable roles in biological control initiatives targeting agricultural and forestry pests [128]. The evolutionary interplay between parasitoids and their hosts has driven the emergence of intricate adaptations that ensure parasitoid success [9]. Key among these adaptations are specialized venom glands capable of synthesizing, storing, and delivering complex biological compounds [3]. In braconid and ichneumonid lineages, females possess calyx cells that produce polydnaviruses (PDVs) [29]. During oviposition, the ovipositor penetrates the host cuticle to deposit eggs and inject an arsenal of parasitic factors, including venom, PDVs, virus-like particles or filaments (VLPs/VLFs), and ovarian secretions, into the host. This induces profound physiological alterations in the host that affect immune function, developmental processes, and metabolic pathways [3,29]. Additionally, certain endoparasitoid taxa produce teratocytes—specialized cells deriving from the dissociation of embryonic serosal membranes during embryonic development—that exert significantly modulate host physiology and biochemistry [107].

In contrast to earlier reviews that primarily focused on individual aspects of host regulation, this article integrates recent advances in endoparasitoid regulation of host immunity, development, and metabolism. It synthesizes findings on venom proteins, PDV-encoded products, and teratocyte-secreted molecules, highlighting how these effectors collectively suppress immune defenses, disrupt developmental programs, and reprogram nutrient metabolism to ensure parasitoid success. Importantly, the regulation of host development, suppression of host immunity, and acquisition of host nutrients are not isolated processes but are closely interconnected. On the one hand, parasitoid-derived virulence factors target multiple aspects of host physiology simultaneously; on the other hand, perturbation of one aspect—such as host development—can exert cascading effects on immunity and nutrient availability. By consolidating these mechanisms, the review establishes a coherent framework for understanding parasitoid host manipulation. This integrative perspective provides insight for future research on virulence factors of endoparasitoid wasps and supports the potential development of sustainable, environmentally compatible pest management strategies.

## Overcoming host defenses as a prerequisite

Insects lack the antibody-mediated adaptive immune system of vertebrates but possess a robust innate immune system that shares fundamental characteristics with vertebrate innate defences [49]. This system enables rapid recognition and elimination of foreign invaders, including parasitoid wasps, through immediate immune activation upon infection. Insect innate immunity is generally divided into cellular and humoral immunity [106]. Cellular immunity involves processes such as proliferation, differentiation, migration, spreading, and adhesion of host hemocytes. Its effectiveness depends on factors including hemocyte subtypes, population dynamics, and compositional fluctuations [106]. Insect cellular immunity encompasses phagocytosis, nodulation, and encapsulation, the latter representing the principal response against large intruders such as parasitoid eggs and larvae [101]. In contrast, humoral immunity relies on phenoloxidase (PO)-mediated melanization and the production of antimicrobial peptides (AMPs), lectins, and other stress-related immune effectors that neutralize foreign agents and contribute to host protection [102]. Cellular and humoral immune responses act synergistically to counter intruders. For instance, upon parasitoid oviposition, the host rapidly mounts a defensive response characterized by the proliferation of hemocytes specialized for encapsulation [137]. These cells migrate to the invasion site and adhere to the parasitoid egg, forming a multilayered, melanized capsule around it [137]. Melanization within this capsule leads to parasitoid death. Parasitoids, in turn, have evolved counterstrategies to subvert or evade host immune responses [88].

### Attenuation of host immunity by hemocyte depletion

In *Drosophila* larvae, the primary hematopoietic organ—lymph gland, is compartmentalized into three distinct zones: medullary zone (MZ), home to stem-like prohemocytes; cortical zone (CZ), which contains differentiated hemocytes, including lamellocytes (a highly specialized immune cell type induced upon infection); and posterior signaling center (PSC), a hematopoietic niche that provides essential signals for the differentiation of lymph gland progenitors into lamellocytes [59,78,141]. The venom of generalist drosophilid parasitoid *Leptopilina heterotoma* (Lh) contains multi-strategy extracellular vesicles (MSEVs), complex secretory structures comprising over 150 proteins (Table 1) [47,120]. Upon oviposition, these MSEVs are delivered into the host and specifically localize to the larval lymph gland, where they disrupt the endomembrane system of macrophage-like plasmatocytes, inducing apoptosis and dislodging the tightly packed PSC cells into isolated singlets or doublets. This disruption impairs the genetic regulatory network responsible for lamellocyte differentiation [95]. Furthermore, Huang et al. identified Lar, a lymph gland apoptosis-related protein, in *L. heterotoma* venom that suppresses host immunity by inducing lymph gland degradation (Table 1). Parasitization by *Lar*-knockdown wasps reduced lymph gland apoptosis, increased lamellocyte production, and enhanced rates of melanotic encapsulation, ultimately resulting in a 2.85-fold decrease in parasitism success [51].

**Table 1:** Endoparasitoid-derived virulence factors and their effects on host hemocyte abundance, encapsulation, and melanization.

**Virulence factors**	**Endoparasitoid**	**Functions**	**Quantitative impact**	**Characteristics**	**Ref.**
MSEVs	*L.heterotoma*	Trigger immunocyte apoptosis and disrupt lamellocyte differentiation	——	Reduce hemocyte abundance	[95]
Lar	*L. heterotoma*	Promotes lymph gland degradation	RNAi *Lar* reduced parasitism success from 77 % to 27 %		[51]
CvBV_26-4	*C. vestalis*	Induces hemocyte apoptosis	CvBV_26-4 disruption reduced apoptotic hemocytes from > 20 % to ∼ 5 %		[127]
CvBV_28-1	*C. vestalis*	Suppresses hemocyte proliferation	RNAi *CvBV_28-1* increased hemocyte density by ∼ 1.5-fold		[131]
TnBVANK1	*T. nigriceps*	Induces hemocyte apoptosis by interacting with Alix	Hemocyte apoptosis increased from ∼ 8 % to > 30 %		[99]
PTP-H2	*M. demolitor*	Induces granulocytes apoptosis	Granulocyte survival reduced by ∼ 70 %		[108]
CpBV-ELP1	*C. plutellae*	Depletes total hemocyte population	Reduced total hemocyte counts by ∼ 3.25-fold		[62]
TnBV1	*T. nigriceps*	Triggers apoptosis-like programmed cell death	Increased hemocyte apoptosis from ∼ 1 % to 27 %		[66]
VPr1/3	*P. hypochondriaca*	Disrupts haemocyte cytoskeleton	——	Disrupt immune cell mobilization	[24,87,97]
P4	*L. boulardi*	Destabilizes the lamellocyte cytoskeleton	Reduced encapsulation rates from 81.2 % to 22.2 %		[64,65]
MmGAP1	*M. mediator*	Disrupts host cellular cytoskeleton via binding key regulators of actin polymerization	RNAi *MmGAP1* reduced wasp egg development to 43.1 % of control		[30]
MmRho1	*M. mediator*	Hijacks cytoskeletal signaling	RNAi *MmRho1* reduced wasp pupation in hosts from ∼ 100 % to ∼ 12.5 %		[125]
Tb4CL4-like	*T. brontispae*	Suppresses host encapsulation via targeting the hemocyte cytoskeleton	Reduced host hemocyte encapsulation rate by ∼ 23 % within 24 h		[148]
MbCRP1	*M. bicoloratus*	Affects cytoskeleton dynamics by inhibition of 42 kDa actin expression	——		[75]
SERCA	*Gana*spi*s* sp.*1*	Blocks plasmatocyte activation and migration by inhibiting calcium signaling	——		[81]
CrCRT	*C. rubecula*	Inhibits hemocyte spreading	CrCRT (12 μg/ml) fully inhibited hemocyte spreading		[146]
PpCRT	*P. puparum*	Inhibits hemocyte spreading	PpCRT (1 μg) reduced hemocyte spreading by > 50 %		[123]
CpBV H4	*C. plutellae*	Interferes with hemocyte spreading efficacy	Decreased host hemocyte spreading by ∼ 70 %		[36]
CpBV-CrV1	*C. plutellae*	Inhibits hemocyte spreading behavior by interacting with GAPDH	1 μmol CpBV-CrV1 completely abolished hemocyte encapsulation		[60,61]
Cp-TSP13	*C. plutellae*	Inhibits hemocyte spreading and protein synthesis	Decreased hemocyte-spreading by ∼ 50 % at 2 μg/μL		[55]
CftICK-I/-II/-III/-IV	*C. flavipes*	Reduce hemocyte spreading and encapsulation and increase larval mortality	Encapsulation index decreased by ca. 40–60 %		[90,92]
Cf4	*C. flavipes*	Impairs hemocyte encapsulation and induces larval mortality	10 μg Cf4 decreased the hemocyte encapsulation index by 1.6-fold		[91]
Glc1.8	*M. demolitor*	Leads to loss of adhesion and phagocytosis in host immune cells	RNAi *Glc1.8* increased host cell attachment from < 25 % to ∼ 90 %		[6,7,116]
WHv1.6	*C. sonorensis*	Prevents immunocytes from spreading or adhering to the parasitoid surface	——		[38]
PpS1V	*P. puparum*	Interacts with host Pro-PAP1 to suppress melanization	PpS1V (0.5 μg) inhibited host hemolymph melanization by ∼ 50 %	Inhibition of PPO activation	[135]
MmvSPN-1/-2	*M. mediator*	Targets serine protease HacSP29 activation to suppress melanization	Knockdown of *MmvSPN-1/-2* increased host PO activity ∼ 4-fold		[150]
CvT-serpins 1/16/18/21	*C. vestalis*	Suppresses melanization by blocking PPO activation	Reduced PO activity by ∼ 25–52 %		[132]
Egf1.0/1.5	*M. demolitor*	Inhibits melanization by disabling PAPs	Egf1.0/1.5 (1 μg) decreased PO activity by ∼ 4–20 fold		[8,73,74]
Vn50	*C. rubecula*	Reduces host PPO proteolytic activity	Reduced PO activity by ∼ 50 % at 0.4 μg/ml		[4,145]
AeSPH1	*A. ervi*	Decreases PPO1 expression to inhibit melanization	RNAi *AeSPH1* decreased mummification rate by ∼ 55 %		[149]
P26	*M.mediator*	Suppresses melanization by targeting the cofactor of PAPs	——		[15,140]
TSVP-8	*C. vestalis*	Suppresses melanization	Decreased melanization ∼ 2-fold at 10 μg/ml		[37]
CvBV_7-1	*C. vestalis*	Reduces host PO activity and inhibits melanization	Disruption of CvBV_7-1 increased PO activity by ∼ 1.7-fold		[130]

Although the anatomical organization of hematopoietic tissues and hemocyte composition varies among insect species, insects rely on specialized hemocytes to execute cellular immune responses. Parasitoids can overcome these defenses by depleting host hemocyte populations. For example, *Cotesia kariyai* infection decreased the circulating hemocyte count in host larvae by ∼78 % within 24 h [114]. This effect is mediated by the combined action of venom and PDVs, which jointly inhibit hemocyte mitosis and induce apoptosis in hematopoietic tissues [114]. In *C. vestalis*, the bracovirus (CvBV) promotes early immune suppression by markedly reducing hemocyte counts, with hemocyte survival in parasitized larvae falling below 70 % [127]. This reduction is mediated in part by the pro-apoptotic effector CvBV_26-4, and functional disruption of this protein alleviates hemocyte depletion while compromising parasitoid larval development (Table 1) [127]. A C-type lectin CvBV_28-1, another CvBV effector, suppresses host hemocyte proliferation and thereby exacerbates the decline in circulating immune cells (Table 1) [131]. RNA interference (RNAi)–mediated silencing of *CvBV_28-1* mitigates hemocyte loss in parasitized *P. xylostella*, whereas ectopic expression of *CvBV_28-1* in *Drosophila* reduces total hemocytes by ∼40 % and lamellocytes by over 60 %. Similar outcomes have been reported with other PDV lineages that employ functionally distinct effectors. In *Toxoneuron nigriceps*, the bracovirus (TnBV) encodes the apoptosis-inducing protein TnBVANK1, an ankyrin-repeat protein homologous to insect and mammalian inhibitors of nuclear factor kappa-B (NF-κB) (IκB), which interacts with Alix (ALG-2-interacting protein X), a co-factor of apoptosis-linked gene protein 2 (ALG-2), and elevates host hemocyte apoptosis from ∼8 % to over 30 % (Table 1) [99]. Silencing of *Alix* completely abolished TnBVANK1-mediated hemocyte apoptosis, confirming the importance of this interaction for its pro-apoptotic function [99]. *Microplitis demolitor* bracovirus (MdBV) expresses the tyrosine phosphatase PTP-H2 which selectively induces apoptosis in granulocytes—the principal phagocytic hemocyte subset responsible for foreign recognition, phagocytosis, and encapsulation initiation—resulting in an approximately 70 % reduction in granulocyte survival (Table 1) [108].

### Encapsulation suppression via immune cell mobilization disruption

For insects, encapsulation is the primary cellular immune response against parasitoid invasion. By targeting cytoskeletal rearrangements essential for hemocyte mobilization, parasitoids can disrupt key hemocyte behaviors, such as migration, spreading, and adhesion, to evade this defense. *Pimpla hypochondriaca* venom contains Vpr1 and Vpr3 proteins that disrupt the cytoskeletal integrity of host hemocytes, inhibiting immune function and promoting cell death (Table 1) [24,87,97]. In *D. melanogaster*, the specialist parasitoid *L. boulardi* employs P4, a venom-derived Ras-homologous GTPase-activating protein (RhoGAP), which remodels actin filament structures via its catalytic RhoGAP domain (Table 1). This remodeling destabilizes the actin cytoskeleton and impairs lamellocyte adhesion, reducing host encapsulation rates from 81.2 % to 22.2 % [64,65]. A more sophisticated strategy is employed by *M. mediator*, whose venom contains MmGAP1, a RhoGAP that interacts with the host GTPases RhoA and Cdc42 (Table 1). The disruption of these GTPases, which are central regulators of actin polymerization, by MmGAP1 impairs hemocyte cytoskeletal organization and suppresses cellular immune responses [30]. In parallel, a guanine nucleotide-binding protein MmRho1 has been identified in the venom of *M. mediator* (Table 1)*.* MmRho1 hijacks cytoskeletal signaling by directly interacting with Diaphanous (Dia), a member of the formin protein family crucial for actin nucleation and elongation [125]. Calcium signaling is as a pivotal upstream regulator of hemocyte activation and mobilization [20,67,79]. In *Drosophila*, plasmatocyte migration toward parasitoid eggs requires a cytoplasmic calcium burst, a process disrupted by the parasitoid *Ganaspis* sp. 1 (G1) through a venom-derived sarco/endoplasmic reticulum Ca^2+^-ATPase (SERCA) pump, which sequesters cytosolic Ca^2+^ in a dose-dependent manner (Table 1) [81]. The resulting Ca^2+^ depletion abolishes Ca^2+^ oscillations, which are periodic fluctuations in intracellular Ca^2+^ concentration essential for signaling and activation, thereby halting plasmatocyte activation and directional migration toward the foreign target [81].

Certain parasitoid virulence factors directly impair the spreading of hemocytes. Two calreticulin proteins, CrCRT from *C. rubecula* and PpCRT from *Pteromalus puparum*, illustrate this mechanism by entering host hemocytes, where they inhibit spreading and consequently prevent encapsulation of parasitoids (Table 1) [123,146]. A CpBV-encoded histone H4 homolog suppresses hemocyte spreading in *P. xylostella*, likely by altering chromatin structure and modulating gene expression (Table 1) [36]. Another CpBV effector, Cp-TSP13, a multifunctional cysteine-rich protein, exerts both immunosuppressive and insecticidal effects by inhibiting hemocyte spreading and reducing host viability (Table 1) [55]. Similar dual-function virulence factors have been identified in *C. flavipes*. For example, Pinto et al. characterized several inhibitor cystine knot (ICK) peptides secreted by *C. flavipes* teratocytes that suppress hemocyte spreading and contribute to host lethality and feeding suppression (Table 1) [90]. More recently, Wang et al. identified Vn1, a peptide highly expressed in the venom apparatus of *Aphidius gifuensis*, that displays oral insecticidal activity [122]. The identification of parasitoid-derived peptides with dual immunosuppressive and oral insecticidal functions will facilitate the development of novel biopesticides.

Parasitoid virulence effectors also target hemocyte–hemocyte interactions. For example, the expression of the *glc1.8* gene from MdBV in hemocyte-like cell lines disrupts cell adhesion and impairs phagocytic capacity (Table 1) [6,7,116]. The *Campoletis sonorensis* ichnovirus (CsIV) encodes a family of 10 cysteine-rich motif (Cys-motif) genes, among which WHv1.6 interacts with host cell membranes and organelles, effectively inhibiting hemocyte adhesion to foreign surfaces (Table 1) [38].

### Targeting PO activation to suppress melanization

In insects, humoral immune responses include melanization, a defense mechanism initiated by the activation of prophenoloxidase (PPO) into PO, triggering a serine protease cascade that converts prophenoloxidase-activating protease (pro-PAP) into PAP, which subsequently catalyzes the hydrolysis of PPO into active PO. The activated PO enzyme facilitates the sequential oxidation of tyrosine to dihydroxyphenylalanine (DOPA) and, eventually, to indole-5,6-quinone intermediates, which polymerize into melanin around invading organisms [83]. The resulting melanin capsule physically isolates the invader within the hemocoel, eliminating it via cytotoxic reactive oxygen species (ROS) or nutrient deprivation.

Endogenous serine protease inhibitors, referred to as serpins, tightly regulate the PO cascade by modulating serine protease activity, preventing excessive or mislocalized activation that could generate ROS and other cytotoxic intermediates and damage host tissues [45,54,83,84]. Parasitoids exploit this regulatory system by delivering serpins that inhibit host serine proteases involved in the PO cascade to suppress melanization. For example, in the pupal endoparasitoid *P. puparum*, PpS1V, a venom-enriched serpin splice variant, binds to *Pieris rapae* pro-PAP1, protecting the developing parasitoid from melanization (Table 1) [135]. Meanwhile, MmvSPN-1 and MmvSPN-2, serpins in *M. mediator* venom, target the host clip-domain serine protease HacSP29 to inhibit PPO activation and AMP transcription (Table 1) [150]. In *C. vestalis*, Wu et al. identified a teratocyte-specific class of serpin genes secreted during parasitism of *P. xylostella*. Among them, CvT-serpins 1, 16, 18, and 21 significantly reduce host PO activity by ∼ 25–52 %, with CvT-serpin 1 directly forming complexes with activated PxPAP1 and PxPAP3, whereas the others likely target alternative proteases within the cascade (Table 1) [132]. PDV-encoded serpins also contribute to melanization suppression. For instance, MdBV encodes Egf1.0 and Egf1.5, which interact with and inhibit the activation and function of the host’s PAP1, PAP3, and serine protease homolog 2 (SPH2) (Table 1). Consistently, RNAi knockdown of *Egf1.0/1.5* completely abolished their anti-melanization activity [8,73,74].

Along with serpins, serine protease homologs (SPHs) also regulate the PO cascade. In *Manduca sexta*, SPH1 and SPH2 are essential cofactors for PAP1-mediated PPO activation [143]. A venom-derived SPH from *C. rubecula* disrupts the interaction among host PPO, PAP1, SPH1, and SPH2, reducing PO activity (Table 1) [4,145]. Similarly, AeSPH1, a homolog protein identified in *A. ervi* venom, suppresses *A. pisum* PPO1 expression, effectively inhibiting melanization and enhancing mummification rates in parasitized hosts (Table 1) [149].

Beyond serpins and SPHs, parasitoid employ additional virulence factors to suppress host melanization. P26, a venom protein found in *M. mediator* and *M. demolitor*, shares homology with viral nuclease toxins and suppresses the cGAS-STING innate immune pathway (Table 1) [15,70]. P26 impairs PO activity by targeting the cofactors of PAPs, reducing melanization responses [140]. TSVP-8, a Kunitz-type venom protease inhibitor identified in *C. vestalis* teratocytes, suppresses melanization (Table 1) [37]. Moreover, the *C. vestalis*-associated bracovirus encodes a family of C-terminal leucine/isoleucine-rich proteins, including CvBV_7-1 and three homologs, that suppress PO activity and host melanization (Table 1) [130]. Expression of *CvBV*_*7-1* in *Drosophila* hemocytes disrupts immune function, and RNAi knockdown of this gene impairs parasitoid development, confirming its critical role in host immune suppression [130].

Collectively, whether through serpins, SPHs, or alternative virulence factors, parasitoids converge on a common strategy: inhibiting the conversion of PPO to active PO to suppress melanization. This conserved targeting mechanism highlights the essential role of the melanization cascade in insect immunity and underscores its value as a focal point of immune manipulation by parasitoids.

### Overcoming host immunity by interfering with immune signaling pathways

**Fig. 1:**
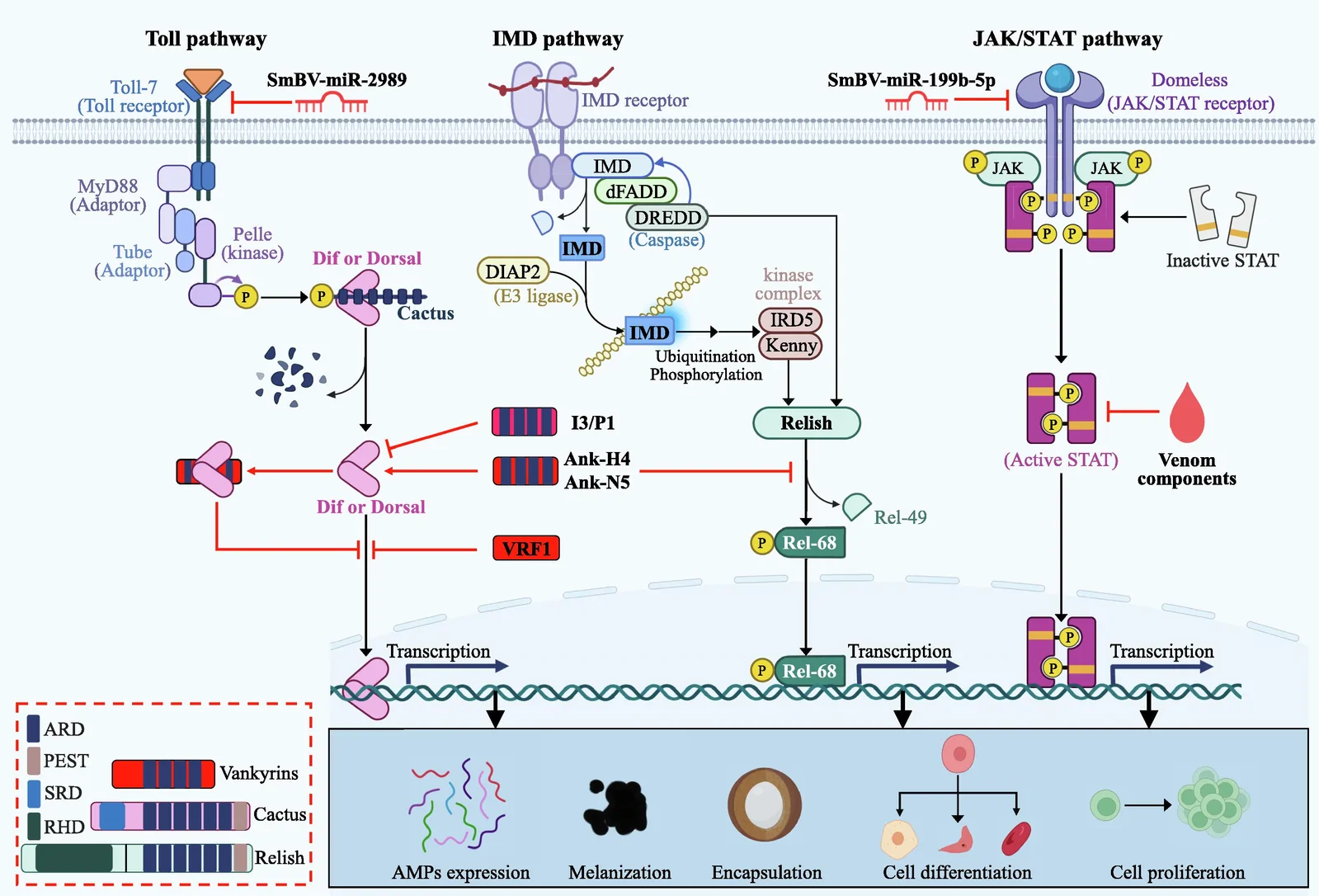
Endoparasitoid-derived effectors interfere with host Toll, IMD, and JAK/STAT immune signaling pathways.

In addition to the melanization response mediated by PO activation, insect humoral immunity critically depends on the regulation of immune effector production via three conserved signaling pathways: the Toll pathway, the immune deficiency (IMD) pathway, and the Janus kinase/signal transducer and activator of transcription (JAK/STAT) pathway (Fig. 1) [19]. These pathways coordinate the expression of AMPs and other immune-related genes, eliciting tailored immune responses to invading pathogens. The recognition of pathogen-associated molecular patterns (PAMPs) by host pattern recognition receptors (PRRs) initiates their activation, triggering complex cascades involving receptor-ligand interactions, enzymatic activation, proteolytic cleavage, and phosphorylation events (Fig. 1) [2,109]. These processes lead to the post-translational modification and nuclear translocation of transcription factors that drive the transcription of immune-responsive genes [2].

The Toll signaling pathway operates via two NF-κB transcription factors, Dorsal and Dif, both of which contain a Rel homology domain (RHD) critical for dimerization and DNA binding [142]. Under basal conditions, Dorsal and Dif are sequestered in the cytoplasm by the IκB-like inhibitor Cactus through its ankyrin repeat domain (ARD). Upon pathway activation, the Toll receptor recruits intracellular adaptor proteins and kinases via its Toll/interleukin-1 receptor (TIR) domain, leading to the phosphorylation and proteasomal degradation of Cactus (Fig. 1) [142]. This releases Dorsal and Dif, causing nuclear translocation and subsequent transcriptional activation of downstream immune genes. The IMD pathway is primarily regulated by the NF-κB transcription factor Relish. Once the pathway is activated, Relish is cleaved by caspase and phosphorylation by kinase complex, producing an N-terminal fragment (Rel-68) that contains RHD, which functions as the active NF-κB transcription factor (Fig. 1) [109]. This fragment translocates into the nucleus to promote immune gene expression, while the C-terminal IκB-like domain remains in the cytoplasm as a regulatory checkpoint [76,109,142]. Similarly, the JAK/STAT pathway activation is characterized by the nuclear translocation of the STAT transcription factor [5]. In *Drosophila*, ligand binding to the JAK/STAT receptor Domeless (Dome) triggers receptor dimerization and activation of the Janus kinase Hopscotch (Hop). Activated Hop phosphorylates STAT, promoting its dimerization and subsequent nuclear import, where it regulates the transcription of downstream immune effector genes (Fig. 1) [5,76,109,142]. These signaling pathways also regulate cellular immunity by controlling the survival, proliferation, differentiation, and encapsulation of immune cells [2]. In *Drosophila*, NF-κB signaling is closely associated with hematopoiesis and the encapsulation of parasitoid eggs [72]. Yang et al. demonstrated that parasitoid infection activates the host JAK/STAT pathway, and its suppression in muscle cells significantly reduces parasitoid egg encapsulation and circulating hemocyte numbers [136].

Given the central role of these pathways in host immunity, parasitoid wasps have evolved diverse strategies to subvert them. For example, although *Stat92E*, the *Drosophila* STAT ortholog, is initially upregulated following parasitoid infection, Brantley et al. demonstrated that virulent wasp species suppress JAK/STAT signaling by targeting key pathway components (Fig. 1) [13]. Tang et al. have identified microRNAs (miRNAs) encoded by *Snellenius manilae* bracovirus (SmBV) that are expressed during parasitism of *Spodoptera litura* larvae. Among these, SmBV-miR-199b-5p suppresses *Dome* expression, thereby impairing JAK/STAT-dependent responses, such as phagocytosis and encapsulation (Fig. 1) [111]. Additionally, SmBV-miR-2989 is predicted to target *toll-7*, a receptor of the Toll signaling pathway, contributing to immune suppression in the host (Fig. 1) [111]. Parasitoid symbiotic bracoviruses (BVs) and ichnoviruses (IVs) encode viral ankyrin (vankyrin) proteins that contain ARDs similar to those of host IκB proteins but lack the signal-receiving domain (SRD) and proline-, glutamic acid-, serine-, and threonine-rich (PEST) motifs required for protein turnover (Fig. 1) [115]. Consequently, vankyrins are insensitive to host signaling factors that normally regulate endogenous IκB phosphorylation, degradation, or cleavage upon immune stimulation. This structural divergence renders vankyrins irreversible inhibitors that sustained suppression of NF-κB–mediated immune responses in host cells. For instance, MdBV vankyrins Ank-H4 and Ank-N5 inhibit Relish processing and bind Dif and Dorsal homodimers, preventing their nuclear translocation and effectively blocking IMD and Toll signaling (Fig. 1) [12,115]. Similarly, CsIV encodes vankyrins including CsIV-P-vank-1 (P1) and CsIV-I2-vank-3 (I3), which act as intracellular IκB-like suppressors of NF-κB, impairing humoral and cellular immune responses (Fig. 1) [44]. Parasitoid venom proteins can directly interfere with immune signaling. Lin et al. identified a metalloprotease homolog VRF1, in the venom of *M. mediator*, which becomes proteolytically activated following host injection. The resulting C-terminal catalytic fragment binds to and cleaves the NF-κB transcription factor Dorsal in *Helicoverpa armigera*, which disrupts Toll signaling and significantly reduces AMP expression and encapsulation efficiency (Fig. 1) [69]. Remarkably, heterologous expression of *VRF1* in the entomopathogenic fungus *Beauveria bassiana* significantly enhances its virulence against *H. armigera* [144].

In summary, immune signaling pathways coordinate humoral and cellular immunity. Parasitoid wasps utilize a diverse array of virulence factors, such as viral miRNAs, IκB mimics, and venom-derived proteases, which cooperate to suppress host immune signaling pathways. By targeting key transcriptional regulators such as Dorsal, Dif, and Relish, parasitoids dismantle host immunity, facilitating successful parasitism in an ongoing evolutionary arms race.

### AMP production for immunosuppression compensation

Parasitoids deploy various effector molecules to suppress host immune defenses, creating a permissive environment for the survival and development of their offspring. However, this immunosuppression compromises the host’s ability to resist opportunistic pathogens, increasing the risk of systemic infection. Such infections threaten the host’s survival and the viability of the developing parasitoid larvae. To mitigate this risk, parasitoids employ chemical arsenals with dual functionality, simultaneously suppressing host immunity and exerting antimicrobial activity that limits microbial proliferation [15]. Proteomic analyses of *M. demolitor* teratocytes have revealed robust secretion of AMPs, which appear to compensate for immune suppression mediated by the symbiotic MdBV [15]. Similarly, the teratocytes of *C. vestalis* produce and secrete a range of serpins. Although some serpins function to inhibit host melanization persistently, others (e.g., CvT-serpin 3, 5, and 15) exhibit novel antibacterial activities [43,132]. These findings suggest that, under pathogenic challenge, immune effectors provide compensatory mechanisms that offset the immunosuppressive effects of other parasitoid-derived virulence factors, which safeguard the host from microbial threats.

Notably, certain parasitoid virulence factors perform multifunctional roles. The C-type lectin CvBV_28-1 suppresses encapsulation by reducing hemocyte numbers and enhances bacterial clearance, protecting the host from secondary infections [131]. By integrating immune suppression with targeted antimicrobial strategies, parasitoid wasps maintain host physiological homeostasis and reduce the risk of fatal microbial infections. This ensures a more stable internal environment conducive to the successful development of their progeny.

### Employing passive strategies for immune evasion

In contrast to actively immunosuppressive strategies, some endoparasitoids utilize passive immune evasion to avoid detection and attack by host immune cells. A well-characterized example involves *L. boulardi*, which secretes a venom protein known as Warm (wasp egg adhesion-related protein with a mucin-binding domain) that facilitates the physical adhesion of the parasitoid egg to host tissues, such as the gut [51]. This adhesion serves as a mechanical barrier that impedes complete hemocyte encapsulation, allowing the parasitoid to evade immune responses and establish successful parasitism [51]. In addition to venom proteins, passive immune evasion involves ovarian secretions and structural proteins expressed on the embryonic surface. In *Macrocentrus cingulum*, the hemomucin protein McHEM localizes to the extraembryonic membrane and prevents encapsulation via extensive surface glycosylation [50]. *M. cingulum* hemomucin possesses a mucin domain composed of polythreonine stretches interspersed with proline residues. This domain harbors the major glycosylation sites of hemomucin, suggesting the O-glycosylation of the mucin domain as a key mechanism underlying immune evasion [139]. Molecular mimicry offers another strategy for immune evasion. In *C. chilonis*, proteomic analyses of ovarian secretions identified five candidate proteins potentially involved in immune evasion, including Crp32B, which significantly inhibits bead encapsulation in a dose-dependent manner [113]. Crp32B shares antigenic similarity with several host proteins, suggesting it functions as a molecular mimic to camouflage parasitoid eggs and facilitate their passive evasion from host immune detection [113].

Importantly, immune evasion and immune suppression are not mutually exclusive. Indeed, many parasitoid–host systems employ both strategies in a complementary manner. For instance, *Hyposoter didymator* eggs avoid encapsulation via protective surface proteins, whereas the developing larvae depend on active immunosuppression mediated by associated PDV to persist in the host hemocoel [28]. These combinatorial tactics reflect the evolutionary sophistication of parasitoid immune modulation, underscoring the dynamic interplay between active suppression and passive evasion in facilitating successful parasitism.

## Growth and development manipulation as the foundation

**Fig. 2:**
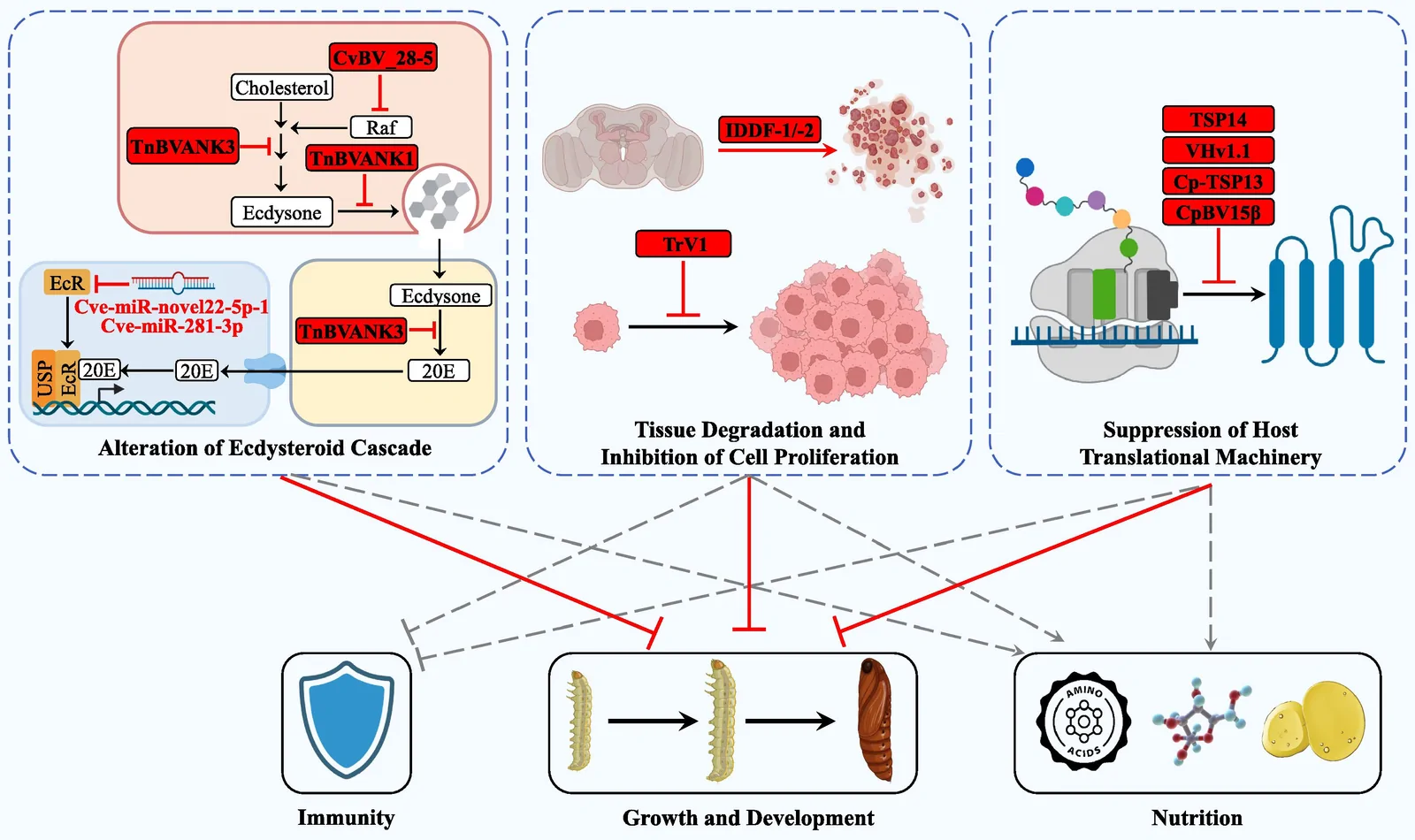
Endoparasitoid-derived effectors manipulate host growth and development through diverse mechanisms. Certain effectors disrupt ecdysteroid biosynthesis or ecdysone receptor (EcR) signaling, blocking molting and metamorphosis. Others degrade tissue or suppress cell proliferation, affecting host growth and development. Some effectors act as translation-inhibitory factors to reduce protein synthesis, contributing to developmental arrest. Alteration of the ecdysteroid cascade may also facilitate nutrient acquisition by endoparasitoids. Virulence factors that degrade host tissues and inhibit cell proliferation suppress host immunity and increase nutrient availability. Inhibition of host protein translation impairs immune responses and reduce host amino acid consumption, thereby promoting nutrient utilization by the developing parasitoid.

Parasitoids can be broadly categorized into two functional types based on their interaction with the host: *conformers*—synchronize their development with host physiology, and *regulators*, which actively manipulate host development to better suit their needs [68]. After overcoming host immune defenses and establishing colonization, parasitoids—particularly regulators—often face developmental asynchrony between their ontogeny and the intrinsic growth trajectory of the host. To resolve this mismatch, parasitoids exert multifaceted control over host development. This is achieved through the alteration of endocrine signaling, inhibition of cellular proliferation, tissue degradation, and suppression of protein synthesis (Fig. 2).

### Alteration of ecdysteroid cascade

Ecdysteroids, primarily ecdysone and its active derivative 20-hydroxyecdysone (20E), are key regulators of insect growth and development, best known for their role in triggering molting. Successful molting depends on precisely timed ecdysteroid pulses that activate specific signaling cascades [117]. Beyond this role, ecdysteroids also regulate metamorphosis, metabolism, and reproduction in insects [117]. For instance, in lepidopteran larvae that have reached the critical size for initiating metamorphosis, the prothoracic gland (PTG) releases a pre-wandering peak of ecdysteroids that enables the final instar larvae to enter the wandering stage in preparation for pupation [85].

Parasitism of *P. xylostella* by *C. plutellae*, which harbors CpBV, markedly prolongs the larval stage and inhibits the larval-to-pupal transition. Functional studies reported that protein tyrosine phosphatases (CpBV-PTPs), encoded by segment S27 of the CpBV genome, disrupt the host endocrine axis by significantly reducing circulating levels of key developmental hormones, including ecdysteroids [56,63]. Similarly, TnBVANK1 impairs ecdysteroid biosynthesis by disrupting the vesicular transport of precursors within PTG cells, resulting in developmental arrest (Fig. 2) [118]. Additional disruption is mediated by TnBVANK3, which suppresses the expression of genes involved in ecdysteroidogenesis and the insulin/TOR signaling cascade, further compromising hormone synthesis (Fig. 2) [52]. Gu et al. have demonstrated that parasitism of *P. xylostella* by *C. vestalis* significantly delays larval molting. This delay correlates with the downregulation of key ecdysteroid biosynthetic genes, including *neverland* and *phantom* [42]. Additionally, CvBV-encoded protein CvBV_28-5 could directly bind to Raf kinase, a central component of the MAPK signaling pathway of the host (Fig. 2) [42]. This interaction disrupts downstream signaling and ultimately suppresses the expression of 20E biosynthetic genes, reducing peak 20E titers in host circulation.

In addition to viral proteins, parasitoid-derived miRNAs have been implicated in hormonal manipulation. Indeed, several miRNAs encoded by CvBV are expressed in infected hosts [129]. Moreover, *C. vestalis* teratocytes can produce and deliver miRNAs into the host. Among these, Cve-miR-281-3p and Cve-miR-novel22-5p-1 significantly reduced ecdysteroid receptor (EcR) expression in *P. xylostella*, delaying molting and metamorphosis by approximately 12 h (Fig. 2) [129]. These findings highlight a multilayered regulatory framework in which parasitoid-derived factors converge to manipulate host endocrine signaling, aligning host developmental trajectories with parasitoid ontogeny.

### Inhibition of cell proliferation and tissue degradation

In some host–parasitoid systems, developmental arrest of the host does not result from hormonal disruption, as juvenile hormone and ecdysteroid titers remain unaffected. Instead, parasitoids achieve developmental manipulation through direct suppression of host cell proliferation and targeted degradation of critical tissues. One of the best-characterized examples is the *Tranosema rostrale* ichnovirus (TrIV), which suppresses host metamorphosis by inhibiting cell proliferation in a dose-dependent manner (Fig. 2) [10]. Among TrIV-encoded virulence factors, TrV1, a 103-amino-acid secretory protein, is abundantly detected in cell culture supernatants and identified as a key mediator of this antiproliferative effect [11]. Functional validation demonstrated that TrV1 expression alone is sufficient to inhibit host cell growth, whereas RNA interference targeting *TrV1* reverses this effect, restoring normal cell proliferation [26]. These findings strongly indicate that TrV1 disrupts host development by directly inhibiting cellular proliferation.

A distinct but functionally analogous mechanism is in the pupal parasitoid *Trichopria drosophilae* (Td), which parasitizes a wide range of drosophilids, including the notably resistant *D. suzukii*. Pang et al. identified two tissue inhibitors of metalloproteinases (TIMPs), Timp1 and Timp2, from the venom, which specifically inhibit host matrix metalloproteinases (MMPs), thereby extending the duration of the host pupal stage to allow sufficient time for parasitoid maturation [86]. A tissue-targeting strategy has been described in *Asobara japonica* (Aj), a koinobiont endoparasitoid that induces developmental arrest by degrading adult precursor tissues [53]. Aj venom triggers imaginal disc degradation (IDD), a process essential for pupal–adult transition, through apoptosis, autophagy, and mitotic arrest, thereby blocking metamorphosis and supporting parasitoid development. Transcriptomic and RNAi analyses identified two venom gland-specific effectors, IDDF-1 and IDDF-2, as key mediators of IDD (Fig. 2) [53]. This finding reveal a distinct strategy employed by koinobiont endoparasitoids: secreting venom proteins that systematically degrade host tissues, enforcing a developmental stasis favorable to parasitoid maturation and emergence.

### Suppression of host translational machinery

As early as the late 20th century, Dahlman et al. discovered that crude preparations of teratocyte-secreted proteins (TSPs) from *M. croceipes in vitro* cultures significantly inhibited the synthesis of developmentally regulated host proteins in *Heliothis virescens* larvae [23]. This translational inhibition led to reduced larval growth, incomplete metamorphosis, and host mortality. Since then, subsequent studies have identified a 13.9 kDa cysteine-rich protein, designated TSP14, as the principal factor mediating teratocyte-induced suppression of host protein synthesis, growth, and development (Fig. 2) [22,96]. Remarkably, transgenic expression of *TSP14* in tobacco (*Nicotiana tabacum*) conferred resistance to both *H. virescens* and *Manduca sexta*, highlighting its bio-insecticidal potential [77]. TSP14 contains a conserved cysteine-rich motif, similar to the cysteine-motif proteins encoded by CsIV, which are known to suppress host protein synthesis [34]. Among them, VHv1.1 exhibited the strongest inhibitory activity, reducing growth in both *H. virescens* and *Spodoptera exigua* [34]. Larvae fed with VHv1.1 displayed delayed development, lower pupation rates, and increased mortality, establishing its role as a potent host translation-inhibitory factor (HTIF) (Fig. 2) [34]. A similar mechanism operates in *C. plutellae* ichnovirus (CpIV), which encodes Cp-TSP13, another cysteine-motif protein identified as an HTIF [55]. Cp-TSP13 is expressed in *P. xylostella* larvae parasitized by *C. plutellae*, where it suppresses mRNA translation in the host fat body, leading to significant reductions in protein synthesis (Fig. 2) [55]. Although the precise mechanisms by which cysteine-motif proteins inhibit translation remain to be elucidated, emerging evidence suggests that they may interfere with the host translational machinery by binding to core initiation factors or disrupting ribosome assembly. For example, CpBV encodes CpBV15β, a viral homolog of eukaryotic translation initiation factors (eIFs) expressed during late parasitism. This HTIF suppresses host mRNA translation by binding eIF4A [93]. Specifically, CpBV15β-mediated translational repression of *P. xylostella* arginine kinase (Px-AK), a key enzyme in energy metabolism that disrupts larval-pupal metamorphosis, demonstrating parasitoids' exploitation of translational control for developmental manipulation (Fig. 2) [93].

Several parasitoid virulence factors exhibit dual functionality, exemplified by the ecdysteroid-modulating proteins CpBV-PTPs and TnBVANK1, as well as the host translation inhibitor Cp-TSP13, which coordinately regulate host development and suppress immune responses.

## Nutritional exploitation as a safeguard

Nutritional dependency is a defining hallmark of parasitism. In endoparasitoids, developing larvae rely entirely on host-derived resources to meet physiological and metabolic needs. To ensure optimal growth and development, parasitoids have evolved precise and multifaceted strategies to manipulate host metabolism, reprogramming it to create a nutritionally favorable environment. Carbohydrates and lipids are primary energy sources, whereas amino acids are fundamental building blocks for growth, collectively determining the success of parasitoid development. The following sections discuss how parasitoids manipulate host amino acid/protein, carbohydrate, and lipid metabolism to achieve nutritional exploitation.

### Enhancement of amino acid and protein availability

Parasitoid wasps, such as *C. chilonis*, are auxotrophic for multiple essential amino acids, having lost the biosynthetic pathways, and therefore depend entirely on host-derived amino acid pools [138]. The absence of any one of these essential amino acids completely arrests the development of *C. chilonis* larvae, underscoring their indispensability for parasitoid growth [138]. To overcome these metabolic limitations, parasitoids employ a two-pronged strategy: suppressing host amino acid consumption and mobilizing amino acids from host protein reserves [133]. The first mechanism involves inhibition of host protein synthesis, as detailed in Section 3.3. This suppression ensures the constant availability of amino acids for parasitoid use, rather than their incorporation into host proteins. Concurrently, parasitoid venoms and teratocytes mobilize host proteins through targeted degradation. Proteolytic enzymes, such as IDDF-1/-2 from Aj, delay host metamorphosis and release amino acid-rich degradation products [53,86]. These proteins thus function as developmental inhibitors and agents of nutritional exploitation, liberating amino acids from host tissues and enriching the hemolymph with digestible nutrients. This creates a nutrient-rich microenvironment that directly supports parasitoid growth while simultaneously depleting host resources.

Parasitoid embryonic teratocytes exhibit remarkable biosynthetic and secretory capacities, with their proteomic output exerting profound regulatory effects on host nutritional metabolism. These specialized cells secrete at least 8 trypsins that systematically dismantle host tissues, converting structural proteins into bioavailable nutrients that fuel parasitoid development, a process that can be suppressed in a concentration-dependent manner by trypsin inhibitors (Table 2) [86]. Beyond tissue catabolism, teratocytes synthesize and secrete macromolecules that mimic the nutrient-rich components of the host hemolymph, such as vitellogenins and storage proteins, which directly serve as pre-digested nutritional sources for parasitoid larvae [107].

**Table 2:** Endoparasitoid-derived effectors and their mechanisms in regulating host development and nutritional metabolism.

**Effector**	**Endoparasitoid**	**Host targets**	**Effects**	**characteristics**	**Ref.**
CpBV-PTPs	*C. plutellae*	Ecdysteroid signaling	Inhibition of larva-to-pupa metamorphosis	Alteration of Ecdysteroid Cascade	[56,63]
TnBVANK1	*T. nigriceps*	Cholesterol trafficking endocytic pathway	Disruption of ecdysone biosynthesis and developmental arrest		[118]
TnBVANK3	*T. nigriceps*	Steroidogenic genes	Blocks the larval-pupal transition		[52]
CvBV_28-5	*C. vestalis*	MAP3K member Raf	Disruption of larval-larval ecdysis		[42]
Cve-miR-novel22-5p-1	*C. vestalis*	EcR	Arrest host growth by modulating EcR expression		[129]
Cve-miR-281-3p	*C. vestalis*	EcR	Arrest host growth by modulating EcR expression		[129]
TrV1	*T. rostrale*	——	Disrupts host development by inhibiting cell proliferation	Suppression of Cell Proliferation and Tissue Breakdown	[10,11,26]
IDDF-1/-2	*A. japonica*	Imaginal disc	Prevent host metamorphosis by degrading the imaginal disc		[53]
TSP14	*M. croceipes*	Protein synthesis process	Suppression of host growth and development	Inhibition of host protein synthesis	[22,96]
VHv1.1	*C. sonorensis*	Translation machinery	Arrests host development		[34,57]
Cp-TSP13	*C. plutellae*	Protein synthesis process	Developmental retardation		[55]
CpBV15β	*C. plutellae*	Px-AK 5′ UTR structure	Inhibits host metamorphosis by suppressing Px-AK translation		[93]
Trypsins	*T. drosophilae*	Host tissues	Dissociate host tissues	Mobilization of host nutrients	[86]
PpAmy3	*P. puparum*	Starch and sucrose metabolism pathway	Hydrolyzes host carbohydrates		[121]
CvT-serpin 8/10	*C. vestalis*	Trehalose and triglyceride metabolic pathways	Regulate host trehalose and lipid levels		[132]
LVL	*Leptopilina* sp.	host lipids	Hydrolyzes host lipids under acidic conditions		[27,58]
Ae-FABP	*A. ervi*	Fatty acids such as myristic acid	Act as a vector transferring FAs from the digestion sites of host lipids to the growing parasitoid larvae		[17]
CvBV_9–2	*C. vestalis*	PxTK signaling	Upregulates host tachykinin signaling to suppress lipogenesis		[126]

### Hijacking host carbohydrate metabolism

During parasitism, parasitoid wasps employ venom and teratocytes to regulate host carbohydrate metabolism. One major strategy involves the secretion of carbohydrate-hydrolyzing enzymes that degrade glycogen into monosaccharides such as glucose and trehalose. For instance, Wang et al. identified an α-amylase, PpAmy3, in the venom of *P. puparum* (Table 2). PpAmy3 is highly expressed in the venom apparatus and transferred into the host hemolymph during oviposition [121]. Metabolomic analyses revealed significant alterations in starch and sucrose metabolism, including changes in mannose, tagatose, glucose-6-phosphate, and trehalose-6-phosphate at varying fold levels. Consistently, RNAi-mediated silencing of *PpAmy3* reduced the size and weight of 1-day-old *P. puparum* larvae, underscoring its role in mobilizing carbohydrates for parasitoid growth [121]. Parasitoids also modulate host endogenous glycosidase activity to enhance carbohydrate availability. In the *C. flavipes–D. saccharalis* system, parasitism rapidly upregulates host α-amylase and trehalase activities by ∼ 1.4- and 1.2-fold within 24 h, respectively [98]. Since *C. flavipes* embryos remain at the egg stage during this early phase, these changes are most likely induced by maternal venom factors rather than larval secretions. A similar trend observed in *Meteorus pulchricornis* parasitism, where two host trehalase genes, *SlTre1* and *SlTre2*, were significantly upregulated in *S. litura* larvae after five days of parasitism [105]. Although the involvement of venom factors in this regulation remains to be confirmed, functional disruptions of trehalase activity via RNAi or treatment with the trehalase-specific inhibitor validamycin A elevated trehalose and reduced glucose concentrations in the host [104,105]. These metabolic imbalances negatively impact parasitoid fitness, as evidenced by prolonged developmental time, reduced emergence rates, and increased developmental abnormalities [104]. These results suggest that enhancing host trehalase expression may be a parasitoid-driven mechanism to ensure conversion of trehalose into bioavailable glucose, sustaining the energy demands of developing larvae.

In addition to venom-mediated regulation, teratocytes play critical roles in reprogramming host carbohydrate metabolism. Wu et al. identified CvT-serpin8 and CvT-serpin10, two serpin-family proteins secreted by *C. vestalis* teratocytes, that can modulate the expression of host metabolic genes. These serpins specifically influence trehalose and triglyceride metabolic pathways, elevating trehalose concentrations in host hemolymph by ∼ 1.4–1.6 fold (Table 2) [132]. Collectively, these findings reveal a comprehensive metabolic hijacking strategy employed by parasitoids, whereby they rewire host carbohydrate metabolism through both direct enzymatic action and indirect gene regulation, securing a sustained and controlled energy supply.

### Mobilization of host lipid metabolism

Lipid reserves represent a vital energy source for parasitoid wasps, particularly during the non-feeding adult stage, when many species lack the capacity for *de novo* triacylglycerol (TAG) synthesis [119]. Lipid acquisition is crucial for sustaining key life-history traits, such as longevity, fecundity, and dispersal; therefore, parasitoids must effectively exploit host lipid metabolism to meet their energetic and developmental needs [100]. Although lipid content in parasitized hosts can vary from unchanged to reduced, previous studies report increased lipid accumulation in the whole body, fat body, or hemolymph of parasitized hosts, reflecting targeted manipulation by the parasitoid.

Parasitoid venom contains a diverse array of enzymes and proteins involved in lipid metabolism [100]. Multiple phospholipases have been identified in venom and are considered key enzymes responsible for lipid degradation. For example, Phospholipase A1 (PLA1) disrupts the phospholipid packaging of cell membranes, leading to cellular lysis, whereas PLA2 hydrolyzes membrane glycerophospholipids, releasing free fatty acids (FFAs) and lysophospholipids [89]. Dong et al. conducted a comprehensive multi-omics analysis of *Leptopilina* parasitoids of *Drosophila* and identified *Leptopilina*-specific venom lipase (LVL), a venom lipase highly conserved across all *Leptopilina* species (Table 2) [27]. Functional characterization revealed that LVL possesses lipolytic activity, with optimal enzymatic function under mildly acidic conditions. Following parasitism, parasitoids induce physiological shifts in the host toward a more acidic environment, thereby enhancing the catalytic efficiency of LVL [27]. This lipase facilitates the hydrolysis of lipid components in the host hemolymph, providing essential nutrients that support parasitoid embryogenesis and promote successful egg hatching. Some venoms also contain enzymes involved in lipid synthesis and storage [21,46]. Although the precise biological roles of these enzymes remain unclear, they may enhance host lipogenic capacity, promoting *de novo* fatty acid and triglyceride production to ensure a continuous lipid supply for parasitoid development. Venom can also reprogram host gene expression associated with lipid metabolism. For instance, parasitism of the cotton aphid *A. gossypii* by *Lysiphlebus japonica* significantly upregulates nearly all key genes in the host’s TAG biosynthesis pathway [134,147]. This transcriptional upregulation correlates with elevated lipogenesis, enriching the host lipid pool available for parasitoid exploitation.

Wang et al. elucidated a molecular mechanism by which parasitoid wasps modulate host lipid homeostasis through PDV-derived effector [126]. *C. vestalis* parasitism induces hypersecretion of tachykinin (PxTK) in the midgut secretory cells of its host, *P. xylostella*, leading to a significant depletion of host TAG reserves, with reductions ranging from 20 % to 50 % across the late third-instar (3L), early fourth-instar (4E), and mid-fourth-instar (4 M) stages. This PxTK-mediated lipid reduction is attributable to CvBV, and further analysis identified the viral effector CvBV_9-2 as the key driver of PxTK expression and lipid consumption (Table 2). Consistently, *CvBV_9-2* was highly expressed in the midgut of parasitized larvae at 3L, 4E, and 4M, while RNAi-mediated silencing of *CvBV_9-2* almost completely restored TAG levels [126]. Notably, parasitized hosts exhibit elevated TAG levels in hemolymph during the late fourth instar, suggesting a stage-specific regulation of lipid metabolism consistent with the changing nutritional demands of the developing parasitoid [126].

Teratocytes released by endoparasitoids secrete carboxylesterases, enolases, and lipases—also commonly found in venom—that contribute to fat body degradation and lipid hydrolysis [15,32,40]. In the *C. kariyai*–*Pseudaletia separata* system, teratocytes attach to the host fat body and secrete collagenases that degrade the surrounding collagen sheath, liberating fat body cells and thereby facilitating lipid acquisition by the parasitoid [82]. Similar functions of teratocytes have been suggested in other parasitoid species as well [94,110]. During the later stages of parasitoid larval development, the number of teratocytes gradually declines, a process attributable, at least in part, to their consumption by the parasitoid [107]. Beyond disrupting fat body integrity to mobilize host lipids, teratocytes themselves store substantial lipid reserves, and their degradation provides an additional energy source [107].

### Facilitating nutrient transport

Nutrient demands of parasitoid offspring vary across developmental stages, accompanied by distinct absorption routes. Studies on *T. nigriceps* and *A. ervi* indicate that nutrients are initially absorbed through the epidermis during early parasitism [18,39,41]. Once a functional gut develops, nutrient uptake shifts primarily to the intestinal epithelium [18]. Parasitoid larvae possess a repertoire of membrane transporters on both body surface and gut epithelia, enabling the translocation of key nutrients such as amino acids and sugars into the hemolymph. For instance, developing *A. ervi* larvae express GLUT2-like and GLUT5-like transporters on the epidermis, which mediate glucose uptake in a concentration-dependent manner, while amino acids are transported by Na^+^-dependent cotransporters [16,18,35].

Complementing these adaptations, venom molecules and teratocyte-secreted proteins play primary roles in promoting lipid transport. Multi-omics analyses have identified numerous lipid-binding proteins in venom, including annexins, apolipophorins, and calreticulin, which are likely involved in membrane transport processes [46,70,71,100,112]. Notably, odorant-binding proteins (OBPs), traditionally associated with olfaction, have also been detected in parasitoid venoms and appear to act as unconventional lipid carriers capable of binding and shuttling fatty acids and esters [25,100,103,124]. Direct mechanistic evidence comes from *A. ervi*, where teratocytes secrete two abundant proteins, p15 and p45, beginning around day 5 post-oviposition, with peak expression during the exponential growth phase of the parasitoid larvae [33]. Among them, p15 has been characterized as an extracellular fatty acid-binding protein (Ae-FABP) with a high affinity for diverse fatty acids (Table 2) [31]. Immunolocalization revealed that p15 accumulates around lipid droplets in host hemolymph as well as along the larval external epidermal layer and within the gut lumen [17]. These findings demonstrate that teratocyte-secreted proteins actively mediate fatty acid transfer from digestion sites of host lipids to the absorptive interfaces of developing parasitoid larvae.

## Conclusion and future perspectives

Parasitoid wasps represent one of the most remarkable examples of evolutionary innovation, exhibiting an exceptional capacity to manipulate host physiology. Their “knowledge” of host physiology is embodied in a sophisticated arsenal that includes venom, teratocytes, and PDVs. These components collectively reprogram host development, immunity, and metabolism to secure parasitoid offspring survival. This review highlights three principal mechanisms by which endoparasitoid wasps exert regulatory control over their hosts: (1) Immune suppression: parasitoids disarm host immune responses by reducing hemocyte abundance, disrupting hemocyte mobilization to inhibit encapsulation, suppressing PO activation to block melanization, and interfering with key immune signaling pathways such as Toll, IMD, and JAK/STAT. (2) Developmental manipulation: to synchronize host development with their ontogeny, parasitoid wasps suppress host metamorphosis by downregulating ecdysteroid titers, inhibiting cell proliferation, promoting tissue degradation, and disrupting protein synthesis, thereby creating a favorable internal environment for larval growth. (3) Nutritional hijacking: parasitoid-derived effectors reprogram host metabolism by directly mobilizing amino acids, carbohydrates, and lipids and indirectly modulating host metabolic enzyme activity to reroute nutrient flows toward the developing parasitoid.

With the rapid advancement of high-throughput omics technologies and biomolecular mass spectrometry, the identification and characterization of virulence factors in this arsenal have become increasingly efficient. Genome sequencing now enables the rapid discovery of PDV-encoded genes. *In vitro* culture, combined with secretome analysis, allows precise profiling of teratocyte-secreted components. Venom component discovery typically integrates differential transcriptomic and proteomic analyses of venom glands. Despite these advances, several technical challenges remain. The extremely low yield of venom and the inability to artificially induce its secretion make complete venom characterization difficult. In addition, the identification of small bioactive compounds in venom remains a major knowledge gap, highlighting the need for further research.

The *Drosophila*-parasitoid system is a classic model for investigating how parasitoid wasps manipulate host physiology. For instance, effectors in *L. heterotoma* venom specifically target the lymph gland, the central immune organ of *Drosophila* larvae, resulting in immune suppression [95]. The venom of *T. drosophilae* directly disrupts adult tissue precursors in *Drosophila*, delaying host development [53]. These findings raise important questions: Do other parasitoid species possess similar “pioneer” effectors? And are there evolutionarily conserved mechanisms targeting key components of host immunity or development? Addressing these requires cross-taxon identification and in-depth functional characterization of virulence factors. Such efforts will be accelerated by integration of multi-omics approaches to achieve a systems-level understanding. Multi-omics integration is transforming parasitoid wasp research. Its shifts the focus from isolated molecular pathways to interconnected, spatiotemporally dynamic networks that reveal how parasitoids manipulate hosts at the whole-organism level. This perspective also helps explain why parasitoid–host interactions vary among species and environments. Ultimately, such knowledge is essential for answering key questions in the field: How do parasitoids coordinate multi-trait host manipulation? Why do certain interactions succeed or fail across species? And how can these mechanisms be harnessed for sustainable pest control?

To date, numerous parasitoid-derived effectors have been identified. Some are peptides with stable tertiary structures that exhibit oral insecticidal activity. Mechanistically, these peptides may act through multiple pathways. One well-documented mechanism involves the disruption of host protein translation. For example, TSP14 and Cp-TSP13 inhibit host translational processes, blocking the synthesis of immune- and development-related proteins and ultimately causing host death. Other peptides, such as the cystine-rich peptides with ICK motifs secreted by *C. flavipes* teratocytes, are hypothesized to target neuronal ion channels. This is supported by their structural similarity to ICK peptides found in arachnid and cone snail venoms, which are known for their potent insecticidal effects. Additional mechanisms may include modulation of intracellular signaling cascades, including stress-response or apoptotic pathways, which could amplify cytotoxicity. These possibilities remain largely unexplored. Clarifying them will be essential for understanding parasitoid biology and for optimizing peptide-based biocontrol strategies. Such mechanistic insights underscore the potential of parasitoid-derived peptides as environmentally friendly biopesticides. Building on this potential, effector genes could be engineered into entomopathogenic microorganisms or transgenic plants to enhance pest resistance. Advances in effector characterization also open new opportunities for synthetic biology applications in pest management. Yeast or bacterial “cell factories” could be engineered to heterologously express venom peptides at scale. Peptide sequences could then be modified to improve stability, oral toxicity, or target specificity. Beyond peptides, synthetic PDV vectors may be designed by replacing native viral effectors with custom gene cassettes. These vectors could enable targeted disruption of pest physiology via engineered parasitoids or direct application as biopesticides. Furthermore, synthetic microbial consortia programmed to produce parasitoid-mimetic effectors in the pest gut may offer sustained pest suppression while maintaining ecological balance. These innovative applications, however, will require rigorous safety evaluation before implementation.

In terms of immune signaling interference, PDV-encoded miRNAs and IκB-related ankyrins can effectively disrupt host immune pathways. However, data on non-PDV-derived effectors remain extremely limited. Lin et al. discovered a venom metalloprotease that suppresses the host Toll signaling pathway by cleaving NF-κB, suggesting that similar signaling-modulating effectors may exist more broadly in parasitoid venoms. Whether teratocyte-secreted factors also participate in immune signaling disruption remains to be determined. Given the critical role of immune pathways in host defense and physiological homeostasis, it is plausible that parasitoid species lacking PDVs have evolved alternative effectors in their venom or teratocyte secretions to modulate these pathways. Compared to immune manipulation, parasitoid regulation of host development and nutrient metabolism requires further research. Based on current findings, parasitoid-induced developmental arrest can be generally categorized into three mechanisms: reduction of ecdysteroid titers, inhibition of cell proliferation and tissue degradation, and disruption of host translational machinery. Some studies also suggest interference through endocrine signals, such as juvenile hormone, though the specific effectors involved remain unidentified. In terms of nutrient regulation, parasitic factors include various metabolism-related enzymes and can alter host gene expression to modulate amino acid, carbohydrate, and lipid metabolism. However, the precise molecular mechanisms by which parasitoids hijack host nutrients remain to be fully elucidated; particularly, how these effectors facilitate the transmembrane transport of nutrients into the parasitoid body remains largely unexplored.

From an evolutionary perspective, parasitoid–host interactions epitomize a molecular arms race. Current evidence suggests that arms races drive rapid diversification: parasitoids evolve novel effectors to bypass host resistance, while hosts evolve counter-adaptations. Phylogenomic analyses across parasitoid taxa and their hosts can help predict evolutionary trajectories of effector repertoires, shedding light on how ecological pressures shape virulence strategies. Such insights will inform sustainable pest control by anticipating the likelihood of host resistance evolution. Additionally, evolutionary predictions will clarify why some parasitoid lineages rely on venom or teratocytes alone—whether due to host-specific selection pressures or evolutionary constraints—and identify untapped virulence mechanisms in understudied taxa.

Looking ahead, climate change introduces a critical layer of complexity. Rising temperatures and altered ecological dynamics can shift host ranges, phenology, and susceptibility, thereby reshaping parasitoid–host interactions. For example, temperature and CO_2_ levels can modify host physiology: elevated CO_2_ often reduces host nutrient quality, which may force parasitoids to evolve enhanced nutrient-extraction effectors or shift to alternative hosts. Similarly, extreme weather events can reduce host population sizes, increasing competition among parasitoids and selecting for more virulent strains. Conversely, climate change may expand the geographic range of invasive pests, requiring parasitoids to adapt to novel host species—with implications for effector diversification and pest control efficacy.

In summary, the application of advanced technologies, from multi-omics to synthetic biology, will revolutionize our understanding of parasitoid-host interactions, while evolutionary and climate-focused research will ensure these insights translate to sustainable pest management. Resolving remaining gaps, such as small molecule effectors characterization, teratocyte roles in immune signaling, and nutrient transport mechanisms, will expand our knowledge of parasitoid biology and unlock new strategies to harness these fascinating organisms in the service of global food security and ecological health.

**Compliance with ethics requirements**

This article does not contain any studies with human or animal subjects.

## Declaration of competing interest

The authors declare that they have no known competing financial interests or personal relationships that could have appeared to influence the work reported in this paper.
